# A Study on DSLM Transporting the Rare Earth Metal La (III) with a Carrier of PC-88A

**DOI:** 10.1155/2018/9427676

**Published:** 2018-06-07

**Authors:** Shibao Lu, Yan Wang, Liang Pei, Wei Li

**Affiliations:** ^1^School of Public Administration, Zhejiang University of Finance and Economics, Hang Zhou 310018, China; ^2^Development and Planning Division, Tongji University, 1239 Siping Road, Shanghai, China; ^3^Key Laboratory of Water Cycle and Related Land Surface Processes, Institute of Geographic Sciences and Natural Resources Research, Chinese Academy of Sciences, Beijing 100101, China

## Abstract

This paper studies transmission behavior of La (III) in dispersed supported liquid membrane (DSLM) of dispersed phase constituted by dispersed supported liquid membrane solution and HCl solution with polyvinylidene fluoride membrane (PVDF) as support and kerosene as membrane solvent, with 2-ethyl hexyl phosphonic acid-single-2-ethyl hexyl ester (PC-88A) and two-(2-ethyl hexyl) phosphoric acid (D2EHPA) as mobile carrier. It also investigates the influence of La (III) transmission by the material liquid acidity, initial concentration of La (III), HCI concentration, membrane solution, and HCI solution volume ratio, resolving agent and carrier concentration, as well as concluding that the optimal transmission and separation conditions are dispersed phase of 4.00 mol/L HCl concentration, 30:30 volume ratio of membrane solution, and HCl solution, within 0.160 mol/L controlled carrier concentration and 4.00 pH value of material liquid. Under the optimal conditions, the La (III) initial concentration of material liquid phase is 8.00 × 10–5 mol/L mol/L, 125 min, and 93.9% migration rate. Under the condition of unchanged acidity of resolving phase, HCL, H_2_SO4, and HNO_3_ as resolving agent, at 125th min, the migration rates of La (III) are 93.9%, 94.0%, and 87.8%, respectively. HCl solution, H_2_SO_4_ solution, and HNO_3_ solution have a certain effect on the La (III) resolution, of which 4.00 mol/L HCl solution and 2.00 mol/L H_2_SO_4_ solution are better. The effect of HNO_3_ is slightly lower than HCl and H_2_SO_4_.

## 1. Introduction

Rare earth metals have wide usage; they cannot only be used alone but also are used in the form of mixed rare earth. Adding a moderate amount of rare earth metals or their compounds in alloy can greatly improve the performance of the alloy; thus, the rare earth elements are also known as vitamin of metallurgical industry. For example, adding some rare earth elements in steel can increase the plasticity, toughness, wear resistance, heat resistance, oxidation resistance, and corrosion resistance [1–4]. Another example is that rare earth metals can be used as a pyrophoric alloy, permanent magnetic materials, superconducting materials, dyeing materials, light-emitting materials, and trace element fertilizer [5–7]. As a result, in addition to being widely used in metallurgy, petrochemical industry, glass ceramic, fluorescent materials, electronic materials, pharmaceutical, and agricultural sectors, the rare earth metal also goes gradually into many areas of modern science and technology. With the wide application of rare earth elements in production and living, it is necessary to separate and enrich rare earth elements, at which a lot of foreign and domestic institutes have been studying recently. The characteristics of liquid membrane extracting rare earth metals comprise a short process, high speed, large enrichment ratio, less reagents, low cost, and wide industrial application prospect. In the system of liquid membrane extracting rare earth metal ion, generally, the organic solvent adopts kerosene or sulfonated kerosene, the carrier adopts LA, P_204_, P_507_, etc., and the internal phase adopts HCI and HNO_3_ [8–11]. Mother liquor leached by the rare earth can be grouped, purified, and separated, etc. according to the need.

In order to overcome these above difficulties in the conventional LM systems, a new liquid membrane technique, namely, dispersion supported liquid membrane (DSLM) [12], was proposed. The DSLM technique is based upon surface renewal, diffusion theory, and our previous work, which also integrates the advantages of fiber membrane extraction process, liquid film transport process, and most of other liquid membrane systems [13]. This is a new type of LM process with several advantages such as increased stability of the membrane, reduced costs, increased simplicity of operation, extremely efficient stripping of the target species from the organic phase by obtaining a high flux, and a higher concentration of the recovered target species in the stripping solution. L. et al. [14] studied the separation of cobalt (II) and lithium (I) in the supported liquid membrane system. He also studied the stirring speed, pH value of material liquid phase, concentration of membrane phase carrier Cyanex272, the concentration of cobalt (II) and lithium (I) in the material liquid phase, and the influence of resolving agent on the two kinds of metal transmission flux. R. et al. [9]. utilized the technology of hollow fiber supporting liquid membrane to extract cadmium in seawater and used ultrasensitive graphite furnace atomic absorption spectrum as detection means to realize selective extraction of cadmium from the water sample. C. J. [15] et al. studied transmission separation of copper, zinc, diamond, and nickel in the supported liquid membrane system, respectively, and used L1X84I, YOPS-99, and Cyanex272 as supported liquid membrane flow carrier, to separate the mixture of copper, zinc, diamond, nickel. Singh et al. [16] reported transmission model of zinc ions in PC-88A-kerosene supporting liquid membrane system. Under the different experimental conditions, a large number of experiments are done to predict the transmission degree of zinc in the supporting liquid membrane system. J. V. et al. [17] reported the transmission of trivalent chromium in the liquid membrane system with the carrier of two (2-ethyl hexyl ) phosphonic acid. T. [18] researched the transmission of divalent and trivalent metal ions as selective migration in the supported liquid membrane system with carrier of new organic phosphonic acid. The research results show that, by using different extraction agents, the mixed ions Cu (I), Co (II), Ni (II), Pb (II), Fe(III), and Cd (II) can be separated well. Sha m si pur et al. studied the transmission separation of silver and mercury in the supported liquid membrane system, improved the ordinary supported liquid membrane system, and set up the system of two film and three chambers, which realized the fast separation of the silver ions and mercury ion. China is rich in rare earth production, many kinds of which need to be separated, purified, and recovered in large quantities; hence the task is very difficult.

The operation of SLM system is simple, which does not require the introduction of expensive surface active agent [10, 19–21], but the membrane solution (organic solvents, extracting agent, and modifying agent) dissolves in water which decreases the stability of membrane and high transmission flux. To settle the existing problem of SLM, some home and abroad studies have been exploring new liquid membrane configuration recently, intending to keep the separation of liquid membrane and overcome the shortcomings [22–24]. So, the terms of “combined liquid brain” and “combined technology” are proposed, which combines a solid film with the liquid film during all kinds of chemical process, effectively overcome the carrier of the supported liquid membrane leaking from membrane phase, and extend the life of the membrane. This work will combine the extraction dispersion technology with the supported liquid membrane and put forward the concept of dispersed supported liquid membrane.

At present, the literatures on dispersed supported liquid membrane separating and migrating rare earth metal are rare. This work mainly discusses and studies the feasibility of dispersed supported liquid membranes separating and migrating La (III), utilizes membrane module design, carrier optimization, and migration rate control to realize the migration and separation of rare earth metals, researches the migration process, and establishes new methods and a new system of dispersed supported liquid membranes migrating and separating rare earth metals, which is expected to be a breakthrough in the industrial application.

## 2. Experiment

### 2.1. Experimental Facility

**Figure 1:**
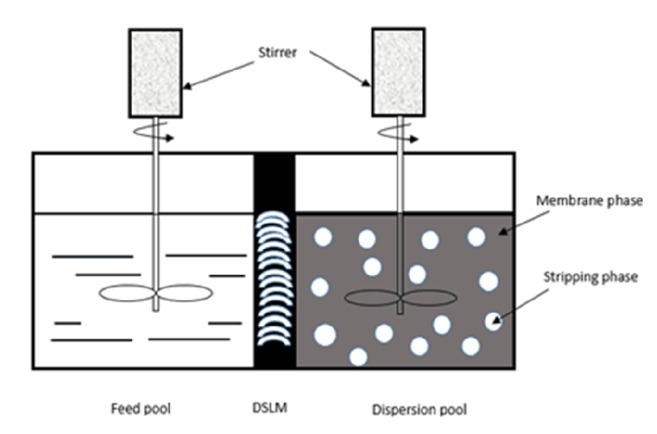
Schematic diagram of DSLM apparatus.

The homemade migration pool of DSLM consists of the material liquid pool, the dispersed pool, and the supported pool, where the capacities of the material liquid pool and the dispersed pool are both 80 mL, equipped with adjustable speed electric blender. The supporting body is hydrophobic porous polyvinylidene fluoride membrane PVDF (Shanghai Yadong Nuclear Grade Resin Co., Ltd.); the diameter of the pore is 0.22 mm, the film thickness 65 mm, the porosity *ε* 75%, the tortuosity factor *τ* 1.67, and the effective area 12 cm2. The experimental apparatus is shown in Figure 1.

UV-1200 type spectrophotometer (Shanghai HuiPuDa Instrument Factory), JJ-1 type precise timed electric blender (Jintan City DanYangMen Quartz Glass Factory), UV-2102PC type ultraviolet-visible spectrophotometer (Unico(Shanghai) Instrument Co., Ltd.), AY120 type electronic scales (Shimadzu), 520MPT atomic emission spectrometer (ChangChun JiLin University Little Swan Instruments Co., Ltd.).

### 2.2. Experimental Method

#### 2.2.1. Preparation of the Solution

They are 1.00 mol/L HAc~ NaAc buffer solution; 1.00 mol/L NaH2PO4~Na2HPO4 buffer solution; 6.00 mol/L HCl; 4.00 mol/L H_2_SO_4_; 1.00 × 10^−2^ mol/L arsenazo III (C22H18As2O14N4S2). Except that the concentration of La (III) is diluted to 1.00 × 10^−2^mol/L by 1.00 mol/L H_2_SO_4_, the concentration of the other standard solutions of various rare earth metal ions should be diluted to 1.00 × 10^−2^ mol/L with 1.00 mol/L HCl.

Membrane solution is made of the flow carrier of PC - 88 A, the concentration of which is diluted to 0.230 mol/L with kerosene.

#### 2.2.2. Operating Steps

The experiment adopts the homemade liquid film transmission device, puts the supported PVDF membrane into the membrane solution to leach and absorb for a certain time (about 3 to 4 hours), then uses the filter paper to suck up liquid on the surface of the membrane, and fixes it in DSLM migration pool. There are two-phase solution in the two slots, respectively, with the isolation of PVDF membrane; one is the material liquid, the other the dispersed phase. A certain amount (5.00 ~ 10.0 mL) of 1.00 × 10^−3^ mol/L solution of rare earth metals and buffer solution (total 60.0 mL) is added to the material liquid phase, while the 60.0 mL mixture of membrane solution and HCl solution are added to the dispersed phase. Start the blender and time it and then take samples of 1.00 mL to 10.0 mL the colorimetric tube from the material liquid phase at a certain amount of time.

#### 2.2.3. Analysis of Samples

Add a moderate amount of the buffer solution and a certain amount of the 1.00 × 10-4 mol/L chromogenic agent arsenazo III (C22H18As2O14N4S2) into the taken samples and dilute it with the deionized water to 10 mL; after 10 min past the chromogenic reaction, use UV-1200 spectrophotometer to measure the absorbance of La (III) at 653 nm.

#### 2.2.4. Analysis of the Results

According to the relationship curve of the absorbance value and the concentration of rare earth metals, the mobility (such as formula (1)) can be calculated. Then the mobility is analyzed quantitatively based on the relationship curve of the absorbance and the concentration of metal. The computation formula is as follows:

(1) $$\begin{array}{c} \eta =\frac{\left(c_{0}-c_{t}\right)}{c_{0}}\times 100\%=\frac{\left(A_{0}-A_{t}\right)}{A_{0}}\times 100\% \end{array}$$

where *η* is for mobility; c0 for metal ion concentration in the starting material liquid (unit: mol/L); ct for metal ion concentration in material liquid phase at time of t (unit: mol/L); A0 for the starting absorbance; At for the absorbance at time of t.

#### 2.2.5. Treatment of Membrane Solution

At the end of the experiment, it is necessary to treat the membrane solution and remove residual rare earth metals in the solution. This experiment adopts 4.00 mol/L H2SO4 to resolve it, and the membrane solution can be recycled.

### 2.3. Experimental Principle

The processes of reaction and the migration of metal ions in the dispersed supported liquid membrane system are as follows.

The La (III) ions in material liquid phase diffused through the water between the material liquid phase and the membrane phase. On the interface of the water phase and the membrane phase, the metal ions La (III) will have the following complex reaction with the carrier of PC–88A (abbreviated to HR):

(2) LaIIIt2++m+n2HR2org=LaRn•mHRorg+nHf+

where the subscript f stands for the water phase, the subscript org for the membrane phase, and (HR) 2 for the extraction agent in the form of dimers in the nonpolar oil.

The metal ions with carrier complex generated by the reaction diffuse from the interface between the material phase and the membrane phase to the inside of the membrane [25] and then diffuse in the membrane phase. At the interface of the membrane phase and the dispersed phase, it takes the following resolving reaction with the resolving agent:

(3) LaRn•mHRorg+nHs+=LaIIIs++m+n2HR2,org

where the right subscript S stands for resolving phase. Due to the fact that the stirring effect causes metal ion with carrier complexes to contact the resolving agent fully, this ensures the continuous extraction and the reverse extraction process and improves the transmission speed of the metal ions and the stability of liquid membrane system. Changing the volume ratio of the resolving agent and the liquid membrane solution can get the resolving solution containing a high concentration of metal ions. Stop stirring; let it stand. The resolving agent contains the high concentration of metal ions and the membrane phase will layer automatically, which is convenient to treat the concentration.

By [6], we know

(4) $$\begin{array}{c} ln\frac{c_{f\left(t\right)}}{c_{f\left(0\right)}}=-\frac{A}{V_{f}}P_{c}t \end{array}$$

where *c*_*f*(*t*)_ and *c*_*f*(0)_ stand for metal ion concentration at time of t and the initial material liquid phase, respectively; A for the effective area of membrane; V for the material liquid volume; Pc for the permeability coefficient of metal ions; t for the transmission time. By measuring the concentration of the metal ions under different conditions, draw relation figure between −ln(c/c0) with t and analyze the influence degree of various factors on the migration rate from the straight slope of the line. The permeability coefficient of the metal ions in the membrane Pc can be indicated by formula (4)

(5) $$\begin{array}{c} P_{c}=\frac{J}{{\left[M\right]}_{f}} \end{array}$$

where J stands for the mass transfer flux of the metal ions; [M] f for the metal ion concentration of the material liquid phase.

When the material liquid phase contains two kinds of ions, if the permeability coefficients of the two kinds of ions in the membrane are different, the ions can be separated by the liquid membrane system. The separation factor is defined as

(6) $$\begin{array}{c} \beta =\frac{P_{c1}}{P_{c2}}=\frac{J_{M1}/{\left[M_{1}\right]}_{f}}{J_{M2}/{\left[M_{2}\right]}_{f}} \end{array}$$

## 3. Results and Discussions

### 3.1. The Influence of pH Value of Material Liquid Phase on the Rare Earth Metal

Known from the mass transfer mechanism of rare earth metal ions in the DSLM, differential concentration of H+ in the material liquid phase and the dispersed phase is mass transfer power of rare earth metals in DSLM [26]. As a result, the higher the pH value of the material liquid, the more conducive to the migration of rare earth metal. But the resolving agent used in dispersed phase is strong acid. So, when the pH value of material liquid is increased to a certain extent, the high strength of H+ differential concentration between the two phases speeds up permeation of the dispersed phase H+ through membrane phase, which seriously affects not only the stability of liquid membrane, but also the migration rate of rare earth metal in the supported liquid membrane. Therefore, the acidity difference between material liquid phase and the dispersed phase is one of the key factors influencing the mass transfer rate of rare earth metals.

What is more, the pH value of material liquid phase can influence the existence of the rare earth metal ions; under the proper pH value, the rare earth metal ions can form complex carrier with the membrane carrier and enter into the liquid film. If the metal ions are transported, the separation effect is well; otherwise, the separation effect is poor. If the pH value becomes too low, the acidity difference between the material liquid phase and the dispersed phase is too subtle and the migration effect will not be satisfying; If the pH of the material liquid becomes too high, it may cause the rare earth metal ion hydrolyse or form hydroxy complex, which affects the migration rate [27]. So, the selection of the material liquid pH plays an important role in metal ion migration.

**Figure 2:**
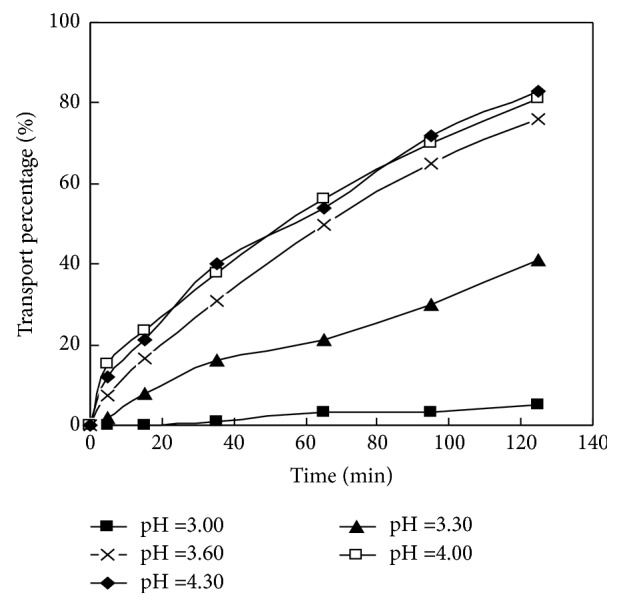
Effect of pH in feed phase on transport of La (III).

The value ratio of the membrane and HCL solution in the dispersed phase of La (III) in DSLM migration system is selected as 30:30; the HCl concentration of the dispersed phase is 4.00 mol/L; the initial concentration of La (III) is 1.00×10-4 mol/L; the concentration of PC–88A in membrane solution is 0.160 mol/L. Under the condition, study the influence of material liquid pH on the migration behavior of La (III) in the DSLM; the experimental results are shown in Figure 2 and in Table 1.

In Figure 2, when the pH values are 3.00, 3.30, 3.60, 4.00, and 4.30, at 125th min, the migration rate of La (III) can reach 4.91%, 38.3%, 75.4%, 81.2% and 82.0%, respectively. When the pH of material liquid is less than 3.00, the difference of the concentrations of H+ in the material liquid phase and the dispersed phase is minor and the migration effect of La (III) is not obvious. When the material liquid pH achieves 3.00, the difference between the concentrations of H+ in the two phases is still small and the migration rate of La (III) is only 4.91%. When the pH value climbs to 3.60, the migration rate increases obviously, twice as much as that of pH 3.30. When the pH value is 4.00, the migration rate increases obviously more than that at pH 3.60; when the pH value is 4.30, the migration rate of La (III) only increases over 0.80% than that of pH 4.00. When pH becomes greater than 4.30, the material liquid acidity is low and high strength of H+ concentration difference between the two phases accelerates the permeation of the dispersed phase H+ through the membrane phase, which influences the stability of liquid membrane and the migration rate of La (III) in DSLM.

Continue to reduce liquid H+ concentration and the La (III) of material liquid hydrolyses, and the solution becomes muddy. From Table 1 we can see that when pH is 3.00, the permeability coefficient of La (III) is 4.47 × 10^−7^ m/s; when the pH increases to 3.60, the permeability coefficient is increased to 1.25 × 10^−5^ m/s, 30 times more than that of 3.00 pH; when pH is 4.00, the permeability coefficient increases to 1.49 × 10^−5^ m/s; on this basis to increase the pH to 4.30, the permeability coefficient is only increased by 3.00 × 10^−7^ m/s when pH is 4.00. Obviously, the best condition is when the liquid pH control is within 4.00.

During the migration process of La (III), the best material liquid pH is selected as 4.00, at 125 min, and the migration rate of rare earth metals in selected condition is 75.2%.

### 3.2. The Effect of HCl Concentration in Dispersed Phase on Rare Earth Metal Migration

The concentration difference of H+ in material liquid phase and dispersed phase is the mass transfer dynamic in DSLM for rare earth metal. We can also change the mass transfer dynamic in DSLM for the rare earth metal via changing the concentration of resolution agent in the dispersed phase based on the determination of the pH value in the material liquid phase. If the concentration of the resolving agent increases, resolving rate increases and the mobility will increase, too. But when the resolving agent concentration increases to a certain extent, the difference of H+ concentrations between the dispersed phase and the material liquid phase becomes too large that the corresponding osmotic pressure difference enlarges; therefore, it is possible that H+ would osmose from the dispersed phase to the material liquid phase. In this case, the membrane solution on the support body and the carrier will run off due to this process, resulting in the phenomenon of decrease in the mobility or instability in the membrane phase. Therefore, we need to study the impact of the concentration of the resolving agent HCl in the dispersed phase on the migration of the rare earth metal.

**Figure 3:**
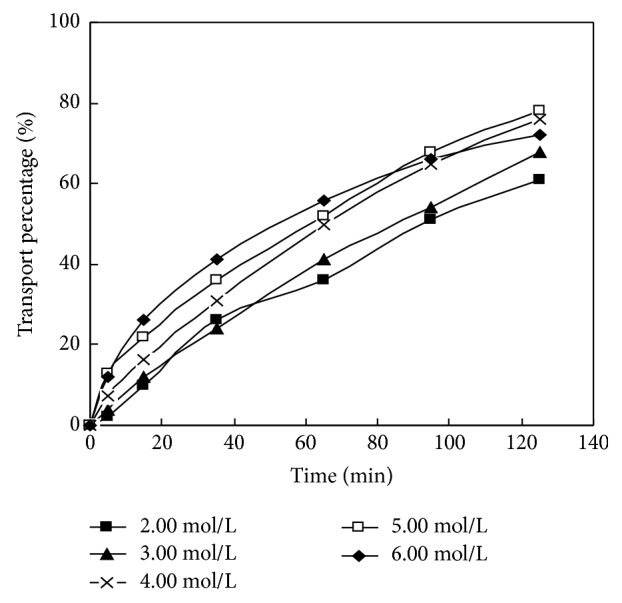
Effect of HCl concentration in dispersion phase on transport of La (III).

The material liquid phase pH of La (III) in DSLM migration system was selected as 3.6, the volume ratio of membrane solution and HCl in dispersed phase was 30: 30, and the concentration of carrier PC-88A in membrane solution was 0.160 mol/L. The initial concentration of La (III) was 1.00×10^−4^ mol/L. In the research on the impact of the concentration of HCl solution in dispersed phase of migration behavior of La (III) in DSLM under this condition, the experimental results are shown in Figure 3 and Table 2.

From Figure 3 we can find that when the HCl concentration of the resolving agent in the dispersed phase increases from 2.00 mol/L to 5.00 mol/L, the migration rate tends to increase, which shows that the more the concentration of the agent becomes, the greater the resolving rate will be, and the migration rate will also be higher accordingly.

From Figure 3 we can see that when the concentrations of HCl are 2.00 mol/L, 3.00 mol/L, 4.00 mol/L, 5.00 mol/L, and 6.00 mol/L, at the time of 125 min, the migration rates of La (III) are 60.3%, 68.1%, 75.2%, 76.0%, and 71.7%, respectively. When the HCl concentration of the dispersed phase is 6.00 mol/L, from 0 to 85 minutes, the migration rate of La (III) is higher than 5.00 mol/L. But after 85 min, the migration rate began to decline, and at 125 min it decreased by 3.50% more than 4.00 mol/L, which attributes to the high acidity of the dispersed phase. Since the large concentration difference of H+ between dispersed phase and material liquid phase leads to large osmotic pressure difference between the dispersed phase and the material liquid phase, the H+ of dispersed phase is likely to permeate to the liquid phase. Meanwhile, the membrane solution on the supporting body is running off. Subsequently the carrier flows away, which results in the reduction of migration rate or instability of membrane phase. When the HCl concentration is 4.00 mol/L, the migration rate significantly presents higher than that of the 2.00 mol/L and 3.00 mol/L of HCl concentration. But when continuing to increase the concentration by 5.00 mol/L, the migration rate increases by only 0.80%. Tables 3-2 also show that the 4.00 mol/L and 5.00 mol/L HCl concentration and the permeability coefficient of the La (III) in DSLM are 1.24 × 10^−5^ m/s and 1.27 × 10^−5^ m/s, with only 3.00 × 10^−7^ m/s difference from each other. From the consideration of acidity control, the appropriate concentration of HCl in the dispersed phase should be 4.00 mol/L.

During migration of La (III), the required best HCl concentration is 4.00 mol/L, respectively, at 125 min, 75 min, 95 min, 130 min, 95 min, and 155 min and the migration rates of six kinds of rare earth metals in the selected condition are 75.2%, 91.2%, 73.5%, 80.6%, 73.5%, and 67.9%.

### 3.3. The Effect of Volume Ratio of the Membrane Solution over the HCl Solution on Rare Earth Metals Migration

Because the dispersed phase is the solution of HCl being dispersed uniformly in the membrane solution, the volume ratio between the membrane solution and the HCl solution directly affects the extraction and resolution rates of rare earth metals [28]. When the total volume of the dispersed phase and the concentration of the carrier and HCl are constant, the higher the ratio of HCl in dispersed phase is, the more unstable the dispersion becomes, which is unfavorable for the migration of rare earth metals. Moreover, with the increase of the HCl solution volume ratio, the membrane solution volume is reduced which means that the carrier number also decreases, so the extraction rate decreases, the resolution rate increases, and the mobility of rare earth metals decreases. When the volume ratio of the HCl solution decreases, the membrane solution volume will increase which means that the carrier number increases, so the extraction reaction rate increases, the resolution rate decreases, and the mobility of rare earth metals increases. When the volume ratio of HCl solution is reduced to a certain extent and then continually reduced, the complex rate of rare earth metals in the material liquid phase is reduced because of the fewer carrier number, as well as the decreased mobility of rare earth metals. The appropriate volume ratio of the membrane solution and HCl solution is the key to improve the mobility.

**Figure 4:**
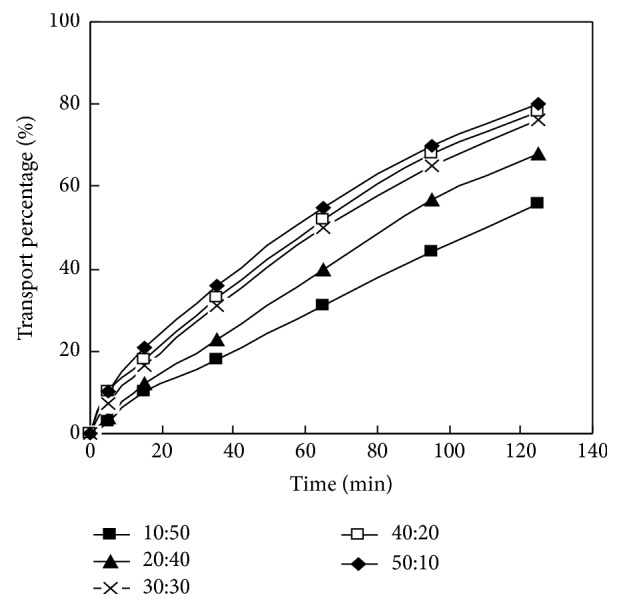
Effect of volume ratio of membrane solution and HCl solution on transport of La (III).

The material liquid phase pH of La (III) in DSLM migration system was selected as 3.6. The initial concentration of La (III) was 1.00 × 10^−4^ mol/L. HCl concentration in dispersed phase was 4.00 mol/L. The concentrations of carrier PC-88A in membrane solution were 0.160 mol/L, 0.160 mol/L, 0.100 mol/L, 0.160 mol/L, 0.100 mol/L, and 0.160 mol/L, respectively. Researching the impact of the volume ratio of the membrane solution over the HCl solution on the migration behavior of La (III) in DSLM under this condition, the experimental results are shown in Figure 4 and Table 3.

It can be seen from Figure 4 that La (III) mobility decreased in turn when the volume ratio of the membrane solution and HCl solution in the dispersed phase changed from 50:10 to 10: 50, and the La (III) mobility were 76.9%, 76.3%, and 75.8% when the volume ratios were 50: 10,40: 20, and 30: 30, respectively, at 125 min. The mobility was only 55.7% and 48.3%, respectively, when the volume ratios were 20:40 and 10:50. Table 3 also shows that the permeability coefficient of La (III) in DSLM increases by only 6.00 × 10^−7^ m/s when the volume ratio increased from 30:30 to 50:10. And the permeability coefficient decreases significantly when the volume ratios are 20:40 and 10:50. It is the traditional SLM system, if the volume ratio was 0:60, the equivalence of the only resolution phase without the membrane solution in the dispersed phase. It can be seen from the results that using DSLM system, when the membrane solution and HCl solution volume ratio in dispersed phase increases from 10:50 to 50:10, the mobility increases, which demonstrates the use of the dispersed phase instead of the traditional SLM resolution phase which helps to improve the mobility of SLM and which proves the superiority of DSLM. The appropriate volume ratio of the membrane solution and the HCl solution should be selected at about 30:30 from economical consideration.

By studying the impact of the volume ratio between the membrane solution and HCl solution in the dispersed phase on the migration behavior of rare earth metals in DSLM system, we can understand the following: the migration of rare earth metals in DSLM system is codetermined by the chemical reaction and the diffusion dynamics and is a dynamic equilibrium process. The entire process is controlled by the chemical reaction, that is, extraction reaction and resolution reaction, when the volume ratio between the membrane solution and HCl solution in the dispersed phase is relatively tiny. According to the principle of the chemical equilibrium, increasing the volume ratio of the membrane solution and HCl solution favors the formation of the carrier complex; therefore the mobility of rare earth metal increases rapidly; but when the volume ratio reaches a certain level, the concentrations of the carrier, rare earth metal, and complex at interface close to saturation. The diffusion process will play a decisive role, so the increase of rare earth metals mobility gradually slows down with the increase of the volume ratio. If the volume ratio continues to increase, the proportion of the resolution agent reduces. And resolution rate will inevitably decline; hence the migration rate also reduces.

In DSLM system and PC-88A as carrier, the best volume ratio of the membrane solution over HCl solution was 30:30 in La (III) migration process and the mobility of rare earth metal La (III) was 75.8% at 125 min under the selected conditions.

### 3.4. The Effect of the Initial Concentration on Rare Earth Metal Migration

In certain DSLM system, if the initial concentration of rare earth ions is too large, the rare earth metal is not fully migrated within a certain period of time [25]; if the initial concentration of rare earth metal ions is too small, the contact rate of metal ions with membrane is very low. These will affect the migration rate. And the measurement range of the instrument for certain elements concentrations should be taken into consideration. Therefore, the initial concentration of material liquid phase has some influence on the migration behavior of rare earth metals.

From (1), the rare earth metal La (III) forms a complex through chemical reaction with the carrier PC-88A at the interface between the material liquid phase and the membrane phase. When the concentration of rare earth metals is relatively low, the balance shifts left and leads to a reduced mobility. Increasing the concentration of rare earth metals, the balance shifts to the right and the mobility increases. But the rare earth metal mobility is also affected by the carrier concentration and the membrane area. When the carrier concentration and membrane area are constant, the number of rare earth metal ions migrating per unit time is certain. So the rare earth metals mobility does not increase with the initial concentration increasing infinitively. When the rare earth metal concentration increases to a certain extent and then as the initial concentration increases, the mobility begins to decline.

**Figure 5:**
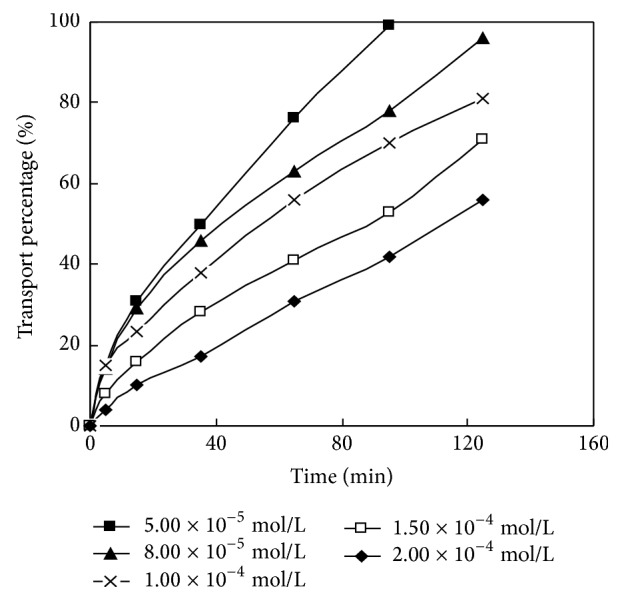
Effect of initial concentrations on transport of La(III).

The volume ratios of the membrane solution and HCl in dispersed phase of La (III) in DSLM migration system were selected as 30: 30, 40: 20, 30: 30, 30: 30, 40: 20, and 40:20. The HCl concentration in the dispersed phase is 4.00 mol/L. The concentration values of carrier PC-88A in the membrane solution were 0.160 mol/L, 0.160 mol/L, 0.100 mol/L, 0.160 mol/L, 0.100 mol/L, and 0.160 mol/L, respectively. The pH values of the material liquid phase were 4.00, 1.00, 5.20, 4.20, 5.00, and 5.10 respectively. Studying the impact of the initial concentration of rare earth metal in the material liquid phase on the migration behavior of La (III) in DSLM under this circumstances, the experimental results are shown in Figure 5 and Table 4.

It can be seen from Figure 5 that La (III) mobility was 93.9%, 81.2% and 67.4%, and 52.8%, respectively, when its initial concentrations were 8.00 × 10^−5^ mol/L, 1.00 × 10^−4^ mol/L, 1.50 × 10^−4^ mol/L, and 2.00 × 10^−4^ mol/L at 125 min. At this time, with the decrease of the initial concentration, the mobility and permeability coefficient are increased. When the initial concentration of La (III) is 5.00 × 10^−5^ mol/L, its migration rate reaches 99.8% at 95 min and no La (III) can be detected at 125 min which has fully migrated. But when La (III) concentration continues to decrease, the balance shifts left and leads to reduced mobility and permeability coefficient.

### 3.5. The Effect of Different Analytical Agents on the Rare Earth Metals Migration

From the mass transfer mechanism of DSLM, the concentration difference of H+ in material liquid phase and dispersed phase is the mass transfer power for metal ions in DSLM [26]. Therefore, when the acidity of material liquid phase and analytical phase are determined, the mass transfer power is generally stable. However, its nature necessarily differs for different types of analytical agents and the ionic environment of analytical phase varies, so the mass transfer power will be slightly different. In this study, the analytical agents are strong acids. HCl, H2SO4, and HNO3 are the strong analytical agents commonly owning different natures. HCl becomes easily volatile and its Cl− is easy to form complex ion with metal ion; SO42− in H2SO4 is easy to form insoluble salts with metal ion; HNO_3_ is oxidizing acid and easy to oxidize organic complexes when its concentrations are high and unfavorable for complex reaction between metal ions and carrier in an oxidizing environment. Therefore, the study of the impact of different analytic agents in dispersed phase on the migration behavior of rare earth is of certain significance.

**Figure 6:**
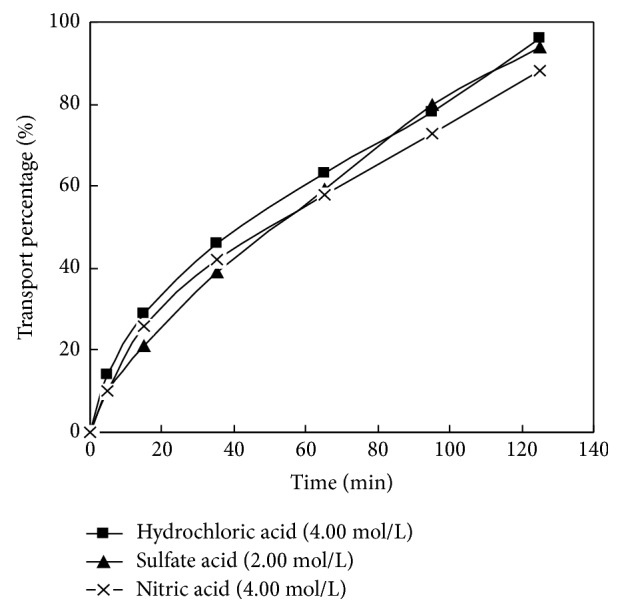
Effect of different stripping agents on transport of La(III).

The volume ratio of the membrane solution and HCl in dispersed phase of La (III) in DSLM migration system was selected as 30: 30. The H+ concentration in the dispersed phase was 4.00 mol / L. The concentration of carrier PC-88A in the membrane solution was 0.160 mol/L. The initial concentration was 8.00 × 10^−5^ mol/L. The pH value of the material liquid phase was 4.00. Researching the impact of different analytical agents in the dispersed phase on the migration behavior of La (III) in DSLM under this condition, the experimental results are shown in Figure 6.

The effects of HCl solution, H2SO4 solution, and HNO_3_ solution on La(III) migration were studied, respectively, in this experiment, keeping the acidity of the analytic phase in dispersed phase stable.

As shown in Figure 6, La (III) mobility values were 93.9%, 94.0%, and 87.8% with HCl, H2SO4, and HNO3 as analytic agent, respectively, at 125 min. It can be seen that the HCl solution, the H2SO4 solution, and the HNO3 solution all have a certain effect on La (III) resolution, in which the 4.00 mol/L HCl solution and the 2.00 mol/L H2SO4 solution were better and the HNO_3_ solution comes the third. This is because of the formation of a stable complex LaCln_3_-n between Cl− in dispersed phase and La (III). Therefore, the 4.00 mol /L HCl was chosen as the analytic agent.

### 3.6. The Effect of the Carrier Concentration on the Rare Earth Metal Migration

The process of DSLM migration of rare earth metals is jointly nominated by the chemical reaction between rare earth metal complexes and the carrier and its diffusion process after bonding [28]. When the carrier is of a low concentration, the mobility is primarily controlled by the chemical reaction between the complex with the carrier. According to the principle of the chemical equilibrium, increasing the concentration of reactants favors complex formation of the carrier material, and therefore the rapider the mobility of metal ions increases, the higher the carrier concentration becomes and the more the adequate response takes place, the higher the mobility turns out. But when the concentration reaches a certain value, the interface concentration is close to saturation, increasing of the carrier concentration contributing to the increasing of the rare earth metal ions mobility levels off gradually. If the carrier concentration is too high and the membrane carrier concentration is saturated, the mobility is primarily controlled by the diffusion of complexes and carrier, which will clog the membrane pores to some extent and lead to the mobility dropping down. Thus, studying the effect of carrier concentration on the rare earth migration is necessary.

**Figure 7:**
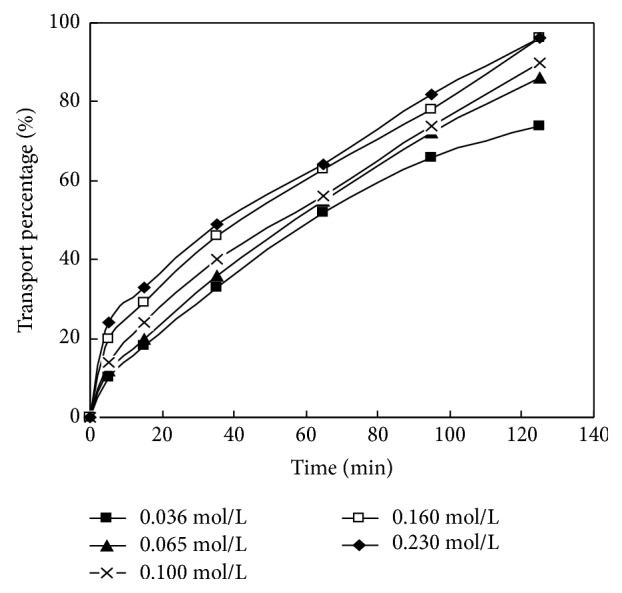
Effect of different carrier concentration on migration of La (III).

The volume ratios of the membrane solution and HCl in dispersed phase of La (III) in DSLM migration system were selected as 30: 30, 40: 20, 30: 30, 30: 30, 40: 20, and 40: 20; the HCl concentration in the dispersed phase is 4.00 mol / L; the initial concentrations were 8.00 × 10^−5^ mol/L, 7.00 × 10^−5^ mol/L, 1.00 × 10^−4^ mol/L, 8.00 × 10^−5^ mol/ L, 8.00 × 10^−5^ mol/L, and 1.00 × 10^−4^ mol/L, respectively. The pH values of material liquid phase are 4.00, 1.00, 5.20, 4.20, 5.00, and 5.10, respectively. Researching the impact of the carrier concentration in dispersed phase on the migration behavior of La (III) in DSLM under this condition, the experimental results are shown in Figure 7 and Table 5.

As can be seen from Figure 7, when the concentrations of PC-88A were 0.036 mol/L, 0.065 mol/L, 0.100 mol/L, 0.160 mol/L, and 0.230 mol/L, respectively, and when the initial concentration of La (III) was 8.00 × 10–5mol / L, the mobility was up to 73.7%, 86.2%, 90.3%, 93.9%, and 94.1% at 125 min. The PC-88A concentration increased; then La (III) mobility increased. When the PC-88A concentration increased from 0.036 mol/L to 0.065 mol/L, the La (III) mobility increased by 12.5%; when the PC-88A concentration increased from 0.065 mol/L to 0.100 mol /L, La (III) mobility increased 4.1%. When PC-88A concentration increased from 0.100 mol /L to 0.160 mol/L, La (III) mobility increased by only 3.6 %. When the PC-88A concentration increased back to 0.230 mol/L, the mobility was 94.1% and only 0.2% higher than that of 0.160 mol /L. When the PC-88A concentration exceeded 0.160 mol/L, the La (III) mobility increased relatively smoothly and was tending towards stability. The La (III) mobility increased rapidly when there was increased carrier concentration in a low concentration range, because the whole process was controlled by the chemical reaction. According to the principle of the chemical equilibrium, increasing the concentration of reactants favors the formation of carrier complex; thus, rare earth metals mobility increases rapidly. But when the concentration reaches a certain level, the interface concentration closes to saturation. And increasing the carrier concentration makes the increasing of mobility of rare earth metals gradually level off. It also can be seen from Table 5 that when the PC-88A concentration increases from 0.036 mol/L to 0.065 mol/L, the osmotic coefficient of La (III) in DSLM increases from 1.19 × 10^−5^ m/s to 1.76 × 10^−5^ m/s by 5.70 × 10^−6^ m/s. When the PC-88A concentration increases from 0.065 mol/L to 0.100 mol/L, the osmotic coefficient increases 4.00 × 10-6 m/s. When the PC-88A concentration increases to 0.160 mol/L, the osmotic coefficient increases by only 3.30 × 10-6 m/s. So with the increasing of the concentration of PC-88A, the increasing of osmotic coefficient tends to level off gradually. On this basis, increasing the PC-88A concentration to 0.230 mol/L, the osmotic coefficient becomes 2.52 × 10^−5^ m/s, only 3.00 × 10^−7^m/s higher than that of 0.160 mol/L. Thus, the concentration of the flow carrier PC-88A is selected as 0.160 mol/L.

Hence, the selected optimum carrier concentration of the dispersed phase in La (III) migration process is 0.160 mol/L and the mobility of rare earth metal La (III) is 93.9% under the selected conditions at 125 min.

## 4. Conclusions

1. The experiments show that DSLM system of (PC-88A-) kerosene—HCl—has a significant role in enrichment and transmission of La (III). The acidity of the material liquid phase, the initial concentration of La (III), the concentration of HCl in the dispersed phase, and the volume ratio of HCI and the membrane solution will affect the transmission of La (III). During the rare earth metal migration process, the most appropriate analytical agent is HCl and the ionic strength of the feed phase has little effect on the migration behavior of rare earth metals in DSLM.
2. The optimum mass transfer conditions for La (III) are that the concentration of HCl in dispersed phase is 4.00 mol / L, the volume ratio of membrane solution and HCl solution 30:30, the carrier concentration 0.160 mol / L, and the pH value of the feed phase 4.00. Under the optimal conditions, the migration rate reaches 93.9% after 125 min when the initial concentration of La (III) in the material liquid phase is 8.00 × 10–5 mol/L.
3. Maintaining the dispersed phase acidity under the same premise, La (III) mobility is 93.9%, 94.0%, and 87.8%, respectively, after 125 min using HCl, H2SO4, and HNO3 as parsing agents. The HCl solution, H2SO4 solution, and HNO3 solution have some effect on La (III) resolving, in which the 4.00 mol/L HCl solution and 2.00 mol/L H2SO4 solution are better for resolving and then comes HNO3.
4. La (III) is the best transmission condition in the separation of La (III) experiment when the concentration of the flow carrier PC-88A is selected at 4.00 mol/L. It proves that the best concentration value of HCl is 4.00 mol/L during the La (III) migration. At 125th min, 75th min, 95th min, 130th min, 95th min, and 155th min, the mobility of rare earth metal La (III) is 75.2%, 91.2%, 73.5%, 80.6%, 70.1%, and 67.9%, respectively, under the selected circumstances.

## Figures and Tables

**Table 1:** Effect of pH in feed phase on transport of rare earths.

Rare earth metal	(min) Migration Time	Item	Results				
La(III)	125	pH	3.00	3.30	3.60	4.00	4.30
		−ln*c*_t_/*c*_0_	0.0503	0.483	1.40	1.67	1.71
		*P*_c_ (m/s)	4.47×10^−7^	4.29×10^−6^	1.25×10^−5^	1.49×10^−5^	1.52×10^−5^

*Note.* Ct and C0 express concentration at time of t and the initial concentration of rare earth metal, respectively, unit: mol/L; Pc expresses permeability coefficient, the unit: m/s.

**Table 2:** Effect of HCl concentration in the dispersion phase on transport of rare earths.

Rare earth metal	(min) Migration n time	item	Results				
La(III)	125	HCl (mol/L) HCl concentration	2.00	3.00	4.00	5.00	6.00
		−ln*c*_t_/*c*_0_	0.924	1.14	1.39	1.42	1.26
		*P*_c_ (m/s)	8.21×10^−6^	1.02×10^−5^	1.24×10^−5^	1.27×10^−5^	1.12×10^−5^

*Note.*C_t_ and C_0_ express concentration at time of t and the initial concentration of rare earth metal, respectively, unit: mol/L; Pc expresses permeability coefficient, the unit: m/s.

**Table 3:** Effect of volume ratio of membrane solution and HCl solution on transport of rare earth.

rare earth metal	(min) Migration Time (min)	item	Data results				
La(III)	125	volume ratio	10:50	20:40	30:30	40:20	50:10
		−ln*c*_t_/*c*_0_	0.660	0.814	1.39	1.41	1.46
		*P*_c_ (m/s)	5.87×10^−6^	7.24×10^−6^	1.24×10^−5^	1.26×10^−5^	1.30×10^−5^

*Note.* Ct and C0 express concentration at time of t and the initial concentration of rare earth metal, respectively, unit: mol/L; Pc expresses permeability coefficient, the unit: m/s.

**Table 4:** Effect of initial concentrations on transport of rare earth.

rare earth metal	(min) Migration Time (min)	item	Data results				
La(III)	125	(mol/L) initial concentration (mol/L)	5.00×10^−5^	8.00×10^−5^	1.00×10^−4^	1.50×10^−4^	2.00×10^−4^
		−ln*c*_t_/*c*_0_	~	2.80	1.67	1.12	0.751
		*P*_c_ (m/s)	~	2.49×10^−5^	1.49×10^−5^	9.97×10^−6^	6.68×10^−6^

Description: “~” represents undetectable, namely, full migration.

**Table 5:** Effect of carrier concentration on transport of rare earths.

rare earth metal	(min) Migration time (min)	item	Data results				
La(III)	125	(mol/L) carrier concentration (mol/L)	0.036	0.065	0.100	0.160	0.230
		−ln*c*_t_/*c*_0_	1.34	1.98	2.33	2.80	2.83
		*P*_c_ (m/s)	1.19×10^−5^	1.76×10^−5^	2.16×10^−5^	2.49×10^−5^	2.52×10^−5^

*Note.* Ct and C0 express concentration at time of t and the initial concentration of rare earth metal, respectively, unit: mol/L; Pc expresses permeability coefficient, the unit: m/s.
